# The Complex Pattern Mismatch Negativity as a Potential Indicator of Psychosis Across all Phases of Illness: A Meta-Analysis

**DOI:** 10.1177/15500594241264870

**Published:** 2024-08-02

**Authors:** Ashley M. Francis, Sydney Slaunwhite-Hay, Kara Dempster, Natalia Jaworska, Philip G. Tibbo, Derek J. Fisher

**Affiliations:** 1 Department of Psychiatry, 3688 Dalhousie University, Halifax, Canada; 2 Department of Psychology, 3684 Mount Saint Vincent University, Halifax, Canada; 3 Institute of Mental Health Research, Affiliated with the University of Ottawa, Ottawa, ON, Canada; 4 Department of Cellular Molecular Medicine, University of Ottawa, Ottawa, Canada

**Keywords:** schizophrenia, complex mismatch negativity, mismatch negativity, psychosis, early phase psychosis, electroencephalogram (EEG), event-related potential, MMN, cMMN

## Abstract

Over the past decade, there has been extensive research on the mismatch negativity (MMN) and its promise as a biomarker of illness in people with schizophrenia (SZ). Nevertheless, when attempting to assess the early stages of illness progression, the utility of MMN has been inconsistent. Recently, researchers have been investigating a more advanced MMN paradigm (the complex MMN [cMMN]) which is believed to index higher-order cognitive processing and has been suggested to be a more effective indicator of the early phases of SZ. The cMMN is defined as a paradigm that relies on alterations within a pre-established pattern of stimuli. In this meta-analysis, we investigated cMMN deficits in individuals with SZ, including an analysis involving those in the first 5 years of illness. Our search also included individuals with bipolar disorder who experience psychosis; however, no related papers were found and thus, no findings are reported. Our findings indicate a small/moderate effect (d = 0.47), suggesting that individuals with SZ exhibit reduced cMMN amplitudes compared to individuals without SZ. Interestingly, this effect seems to be more pronounced in individuals within the first 5 years of their illness (d = 0.58), suggesting that cMMN might be a more sensitive biomarker in the early phases of SZ compared to traditional paradigms.

## Introduction

### Mismatch Negativity Deficits in Schizophrenia

The mismatch negativity (MMN) is an electroencephalography (EEG)—derived event-related potential that is proposed to index automatic pre-conscious auditory change detection in the brain,^1^ and reflects an automatic echoic memory comparison process.^2,3^ The auditory MMN is elicited by presenting a stream of auditory stimuli; the brain holds these sounds in memory and creates a predictive model of the next tone. An MMN is elicited when the predicted and presented tone are not congruent, or are mismatched.^4^ For a full review of the predictive coding model, see Kirihara et al, 2020 and Millidge et al, 2021.^5,6^

The traditional paradigm used to elicit the MMN is often referred to as the oddball task. It is an auditory task that consists of a stream of tones presented at a consistent pitch. A deviant tone is then introduced to break this pattern, which elicits an MMN waveform. The deviant tone typically deviates in terms of pitch, location, duration, gap, or intensity relative to the more frequent sound (ie, the standard). Previous studies consistently show that individuals with schizophrenia (SZ) experience decreases in MMN amplitude, with moderate to large effect sizes.^7–10^ The most consistent findings come from the duration deviant whereby smaller MMN amplitudes are reported, nevertheless, smaller effect sizes (ie, less of a difference between clinical and control groups) are reliably observed in the early phase/first-episode psychosis (FEP) population.^8,11^

Multiple studies have found significant correlations in SZ and other disorders with psychotic features (such as bipolar disorder) between duration MMN amplitude deficits and longer illness duration,^12,13^ worse social and occupational functioning,^14^ more severe cognitive symptoms (such as impaired working memory and verbal fluency),^15,16^ and increased psychiatric symptoms (ie, positive and negative symptoms).^14,17–20^ As such, the MMN may serve as a valuable biomarker for illnesses that express psychotic symptoms rather than diagnostic specificity.

### Complex MMN

Recently, there has been an interest in utilizing MMN paradigms that require more complex neural resources to investigate if the MMN could be used as an indicator of early phases of illness progression (ie, identifying individuals based on a genetic or clinical high risk or within the first few years of illness).^21^ Specifically, this has focused on paradigms that employ a deviant varying from a standard in multiple physical dimensions or violating an established pattern elicit a complex pattern MMN (cMMN). It is hypothesized that cMMN generation relies on higher-order cognitive processing involving the bilateral dorsolateral prefrontal cortex^22^ in addition to the primary auditory cortex,^7,23,24^ whereas the generation of the MMN relies primarily on the primary auditory cortex,^25^ with some contributions from a frontal generator.^23,26^

Due to the computational complexity and demand on higher order processing that the cMMN generation requires, it is hypothesized that the cMMN will deteriorate earlier in the progression of the illness compared to the traditional MMN. Therefore, the cMMN may be a more sensitive marker of early illness profiles. Currently, there are several cMMN paradigms using different methodologies to elicit the cMMN, such as complex pattern paradigms including the introduction of a missing tone,^27^ or the repetition of a tone in a previously established pattern.^28,29^

Unlike the traditional oddball paradigms that consistently show deficits in MMN amplitude in individuals with SZ^7,10,30^ the same consistency has not been observed for the cMMN. While some paradigms report alterations in cMMN amplitudes in individuals with SZ^31^ others find no group differences,^28^ which may be related to the variability within the paradigms. A previous meta-analysis by Avissar et al (2018) provided a brief insight into the cMMN paradigms. Overall, the meta-analysis indicated that their complex abstract/pattern paradigms showed slightly better performance compared to the traditional oddball paradigms (d = .75, d = .59 respectively). They go on to say that while the complex paradigm might excel in testing specific neuroscientific hypotheses, it does not demonstrate superiority over the traditional oddball paradigm in distinguishing individuals with SZ from those without.^7^ The paradigms utilized by Avissar et al (2018), however, involved complex sensory stimuli presented in an oddball paradigm, rather than employing a complex pattern MMN paradigm (cMMN). As such, these findings do not sufficiently represent those that may be observed with complex pattern paradigms only.^7^ Complex sensory paradigms rely on changes in sensory features as opposed to complex pattern paradigms, which rely on predictors based on higher-order cognition (eg, Gestalt principles).

### Current Study

The complex pattern MMN is believed to involve more complex computational brain processing; as such, it may be more sensitive to detecting disease onset at an earlier time. The aim of this meta-analysis was to examine whether the cMMN could serve as a reliable biomarker for both the early phase, as well as more established, SZ. Additionally, diagnostic specificity was examined by attempting to assess cMMN in BD.

## Methods

For this meta-analysis, complex MMN paradigms were defined as paradigms that rely on alterations within an already-established pattern of stimuli to trigger a cMMN response. These paradigms are thought to rely more heavily on higher order cognitive processing compared to the traditional paradigms.

### Literature Search

**Figure 1.**
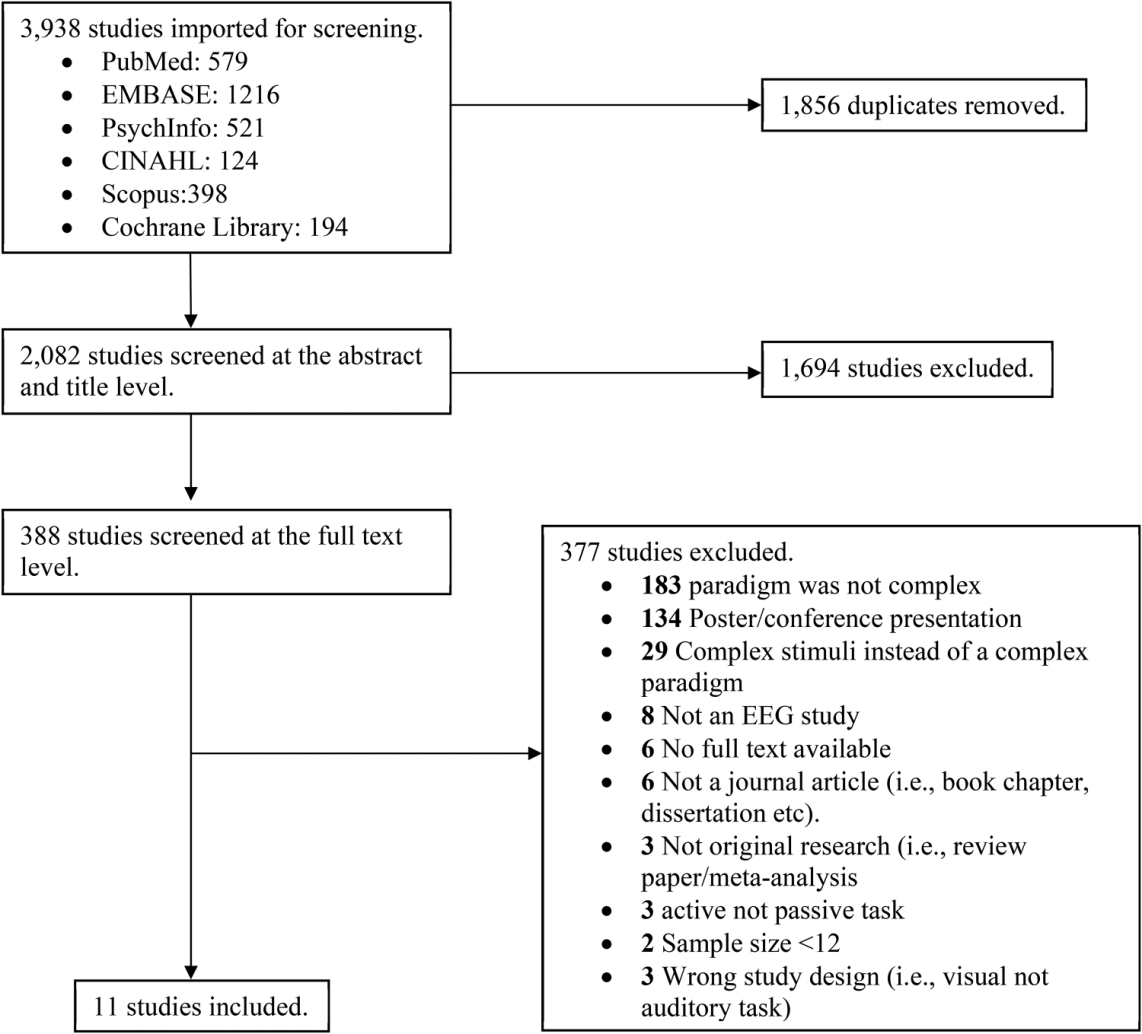
PRISMA diagram. PRISMA: Preferred Reporting Items for Systematic Reviews and Meta-Analyses (PRISMA) diagram represents the number of studies included and excluded at each stage of the screening process.

A literature search was conducted following best practices for conducting systematic literature reviews^32,33^ and in accordance with the Preferred Reporting Items for Systematic Reviews and Meta-Analyses guidelines.^34^ The search terms and search strategy were generated in consultation with an evidence synthesis librarian at Dalhousie University. The search terms were designed to cover the broad fields of psychosis (eg, psychosis, SZ, psychotic disorder, BD) and EEG (eg, complex MMN, cMMN; see Table 1). Terms within the same category were combined using OR while terms from different categories were combined using AND. Five databases were searched (ie, PsycInfo, PubMed, EMBASE, CINAHL and Web of Science) and 3550 papers were identified. For a detailed breakdown of the number of papers yielded by each search, refer to Figure 1.

**Table 1.** Search Terms Used in the Meta-Analysis.

	Psychosis	Imaging
Search terms	Psychosis	Complex MMN
	Schizophrenia	Mismatch negativity
	Schizo*	MMN
	Psychotic disorder	cMMN
	Severe mental illness	Complex mismatch negativity
	Serious mental illness	
	Mental illness	
	Bipolar disorder	
	Bipolar disorder 1	
	Manic depression	
	Bipolar affective disorder	
	Bipolar depression	

MMN, mismatch negativity; cMMN, complex mismatch negativity. * Signifies a truncated search term.

Following the removal of duplicates (1856), there were 1694 papers eligible for the title and abstract screening phase. Two reviewers (A.M.F., S.S.H) independently screened the titles and abstracts for relevance to determine if they met the criteria for full-text review, based on strict inclusion/exclusion criteria (see Table 2). Conflicts were resolved through consensus with another member of the review team (D.J.F.). A total of 374 papers underwent full-text review, and 11 studies were extracted and included in this meta-analysis.

**Table 2.** Inclusion Criteria for the Meta-Analysis.

	Inclusion Criteria
Psychosis	Must include those within the early phase or chronic SZ or BP— confirmed by clinical charts or questionnaires during the study. A comparison group must be present of those without a diagnosed psychotic disorder.
EEG	Must include the complex MMN paradigm.
Overall	Sample size > 12. Based on best standards high-impact neuroimaging research. No case studies. Published in English. Must have been published after 1990^1^ (first cMMN study). Human studies (no animal models). Must be original research (no reviews/meta-analysis). No conference abstracts/posters or book chapters. Must have effect sizes reported or data available to calculate them. Must be published in a peer-reviewed journal.

1 ^66^

SZ, schizophrenia; MMN, mismatch negativity; cMMN, complex mismatch negativity; EEG, electroencephalography.

#### Primary Outcome

The primary outcome of this systematic review was to assess cMMN sensitivity in early and chronic SZ and BD. Specifically, we examined if differences between clinical and control groups can be observed, including the magnitude of the effects at a group level.

#### Data Extraction

The first author independently extracted the data necessary for this review, including participant characteristics, sample sizes, and mean and standard deviations of the main amplitude findings at the site of maximal amplitude (see Tables 3 and 4 for more details).

**Table 3.** Extracted Data from the 11 Papers Included in the Analysis.

Author	Year	N		Sex (F)					
		SZ	HC	SZ	HC	Age (SD)	Amplitude (mean ± SD)	Reference Used	Complex Paradigm Used
Mori et al	2021	17	15	17 (0)	15 (0)	-	SZ: −0.31 (1.89) HC: −1.07 (1.38)	Nose	Missing stimulus paradigm—missing sixth stimuli
*Rudolph et al	2015	13	15	9 (4)	9 (4)	SZ: 24.8 (1.3) HC: 26.4 (2.0)	SZ: 0.46 (1.37) HC: −1.43 (1.36)	Nose	Missing stimulus paradigm with either a missing fourth or sixth stimuli
Salisbury et al	2018a	20	22	15 (5)	14 (8)	SZ: 32.6 (6.4) HC: 33.4 (11.3)	SZ: 0 (1.4) HC: −1.1 (1.2)	Nose	Ascending pitch paradigm
Salisbury et al	2018b	24	21	16 (8)	15 (6)	SZ: 36.5 (8.1) HC: 33.9 (8.8)	SZ: −0.4 (1.6) HC: −1.4 (1.6)	Nose	Ascending pitch paradigm
*Salisbury et al	2018c	21	21	15 (6)	14 (7)	SZ: 21.9 (4.8) HC: 22.2 (4.8)	SZ: −1.4 (2.2) HC: −1.1 (1.9)	Nose	Ascending pitch paradigm
Haigh et al	2018	27	26			-	SZ: 0.2 (1.73) HC: 0.08 (1.76)	Nose (then digitally to averaged mastoid)	Alternating ascending pitch (two up one down)
Haigh et al	2016	27	27	16 (11)	14 (13)	SZ: 36.0 (7.9) HC: 32 (11.2)	SZ: −0.5 (1.15) C: −1.05 (1.15)	Nose (then digitally to averaged mastoid)	Additional tone
Haigh et al	2017a	27	28	18 (9)	18 (10)	SZ: 36.1 (8.2) HC: 32.4 (10.5)	SZ:—0.24 (0.35) HC: −0.78 (0.35)	Nose (then digitally to averaged mastoid)	Ascending pitch paradigm
Haigh et al	2017b	24	26	17 (7)	16 (10)	SZ: 35.5 (8.3) HC: 32 (10.4)	SZ: −1.7 (1.86) HC: −2.67 (2.14)	Nose (then digitally to averaged mastoid)	Ascending and descending pitch paradigm
Ells et al	2018a	15	12	9 (6)	8 (4)	SZ: 24.6 (1.0) HC: 26.4 (2.0)	SZ: −1.7 (1.47) HC: −2.67 (1.45)	Nose	Pattern paradigm with repeating pure tones
*Ells et al	2018b	6	12	3 (3)	8 (4)	SZ: 23.6 (1.5) HC: 26.4 (2.0)	SZ: −1.27 (1.86) HC: −2.67 (1.45)	Nose	Pattern paradigm with repeating pure tones
Ells et al	2018c	9	12	6 (3)	8 (4)	SZ: 25.2 (1.4) HC: 26.4 (2.0)	SZ: −2.32 (1.42) HC: −2.67 (1.45)	Nose Nose	Pattern paradigm with repeating pure tones
Alain et al	2002	17	17	9 (8)	9 (8)	SZ: 28.3 (6.9) HC: 27.9 (7.6)	-	Cz during recording then average referenced in offline processing	Paradigm with differing frequency and location of the tones
Salisbury et al	2016	14	16	9 (5)	10 (6)	SZ: 34.5 (12.8) HC: 35.4 (11.6)	SZ: 0.29 (1.22) HC: −0.77 (1.35)	Nose (then digitally to averaged mastoid)	Missing stimulus paradigm with either a missing fourth or sixth stimuli
*Salisbury et al	2020	22	22	15 (7)	13 (9)	SZ: 22.0 (4.8) HC: 23.6 (7.8)	SZ: −0.1 (1.5) HC: −1.1 (1.6)	Nose (then digitally to averaged mastoid)	Additional tone (fourth tone)
Davalos et al	2005	15	13	-	-	SZ: 39.3 (10.7) HC: 34.3 (10.6)	SZ: −1.44 (0.83) HC: −1.95 (0.91)	Nose	Altered ISI between tones

* = Study includes individuals within the early phase of psychosis.

a, b, c = Denotes a sub-study with separate analysis with unique participants pulled from the same published paper.

SZ, schizophrenia.

**Table 4.** Data Extracted from Papers Including Illness Duration and Medication Usage.

Author	Year	Illness Duration	Medication at Time of Testing
Mori et al	2021	N/A	Antipsychotic Medication
*Rudolph et al	2015	6 months—5 years	Antipsychotic medication or adjust drugs
Salisbury et al	2018a	At least 5 years illness duration, or hospitalized at least three times	Medicated and moderately symptomatic
Salisbury et al	2018b	At least 5 years illness duration, or hospitalized at least three times	FEP had less than 2 months of lifetime antipsychotic medication exposure and 8 FEP were unmedicated at the time of testing.
*Salisbury et al	2018c	Within 2 months of their first clinical presentation of FEP	A FEP had less than 2 months of lifetime antipsychotic medication exposure and 8 FEP were unmedicated at the time of testing.
Haigh et al	2018	At least 5 years illness duration, or hospitalized at least three times	Antipsychotic medications
Haigh et al	2016	At least 5 years illness duration, or hospitalized at least three times	Medicated and moderately symptomatic
Haigh et al	2017a	At least 5 years illness duration, or hospitalized at least three times	Antipsychotic medication
Haigh et al	2017b	At least 5 years illness duration, or hospitalized at least three times	Antipsychotic medication
Ells et al	2018a	Between 2 and 5 years of illness	Atypical anti-psychotics or adjunct medication including clonazepam, escitalopram, lamotrigine, prazosin, and venlafaxine.
*Ells et al	2018b	Average illness duration of 2.95 years	Atypical anti-psychotics or adjunct medication including clonazepam, escitalopram, lamotrigine, prazosin, and venlafaxine.
Ells et al	2018c	Average illness duration of 4.27 years	Atypical anti-psychotics or adjunct medication including clonazepam, escitalopram, lamotrigine, prazosin, and venlafaxine.
Alain et al	2002	Symptoms began 2-15 years before the study	Atypical anti-psychotics
Salisbury et al	2016	N/A	Medicated and moderately symptomatic
*Salisbury et al	2020	FEP participated within the first 2 months of their clinical presentation and had less than two months of use of antipsychotic medication	Five were unmedicated and the rest were on antipsychotic medication for less than 2 months
Davalos et al	2005	N/A	Atypical anti-psychotics and one participant was unmedicated.

FEP = First-episode psychosis.

* = Study includes individuals within the early phase of psychosis.

a, b, c = Denotes a sub-study with separate analysis with unique participants pulled from the same published paper.

### Quality Assessment

To evaluate the potential systematic risk of bias in the review, both the first and second authors independently assessed each of the included 11 articles. A Cochrane Risk of Bias score was assigned, considering factors such as completeness of outcome data, presence of selective outcome reporting, and other sources of possible bias for each article.^35,36^ Each category was rated as low, high or unclear of risk of bias.

### Data Encoding

Variables of interest, including mean (M), standard deviation (SD), and sample size (N), to calculate effect sizes for MMN amplitudes (in μV) in clinical participants (SZ or BD) and healthy controls were exported for analyses. Whenever possible, we reported findings specifically for MMN amplitudes at frontal and midline sites, particularly Fz, as this is known to be the site of the maximal amplitude.^3,37^ In some cases, the mean included data from all scalp sites or an average of the midline or frontal, frontocentral, and/or central scalp sites, when this was the case the mean provided was used. Where multiple experiments or different paradigms were reported within the same manuscript, we treated these as separate studies, thus, yielding (n = 16) unique samples that were included in our meta-analysis. This can be seen in Tables 3 and 4 denoted with an a, b, or c depending on the number of sub-studies included. There were six studies^11,38–42^ that included multiple deviants that were tested on the same group of participants; for this analysis, an average across all deviant types was generated for controls and the clinical sample. For each study, we also recorded participant age, sex, illness duration, medication status, and complex paradigm type/deviant type used, and MMN quantifications.

### Statistical Analysis

Summed Cohen's d effect sizes were generated from mean difference scores using a custom R script and were used to compare cMMN amplitudes between patient and control conditions. Cohen's d effect sizes can be interpreted as small (d = .2), moderate (d = .5), and large (d = .8). A meta-analysis using the random effects and inverse-variance weighting method was performed utilizing the R meta package (R Core Team. R: A Language and Environment for Statistical Computing. 2023 Retrieved from https://www.R-project.org/). The degree of heterogeneity among studies was evaluated using the *I*^2^ statistic and its corresponding *P*-values.

## Results

Although we intended to include studies with participants experiencing various forms of psychosis, such as SZ and BD, it is important to note that the studies included in this analysis solely focused on individuals diagnosed with SZ, as no eligible papers assessed cMMN in individuals with BD. Therefore, for the remainder of this paper, when referring to the patient sample, we are specifically referring to clinical participants diagnosed with SZ. In total, our sample consisted of 603 participants (SZ = 298; HC = 305), with 175 female participants, n = 82 of them being clinical participants. More detailed information on the demographics of these participants can be found in Tables 3 and 4.

A total of five unique paradigms were identified in this analysis. These included studies using a missing tone,^42,43^ an additional tone,^40,44^ complex changes in pitch,^11,39,45^ repetitions in pre-established patterns,^28^ alterations of the interstimulus interval,^38^ and alterations of tone location and pitch.^46,47^

**Figure 2.**
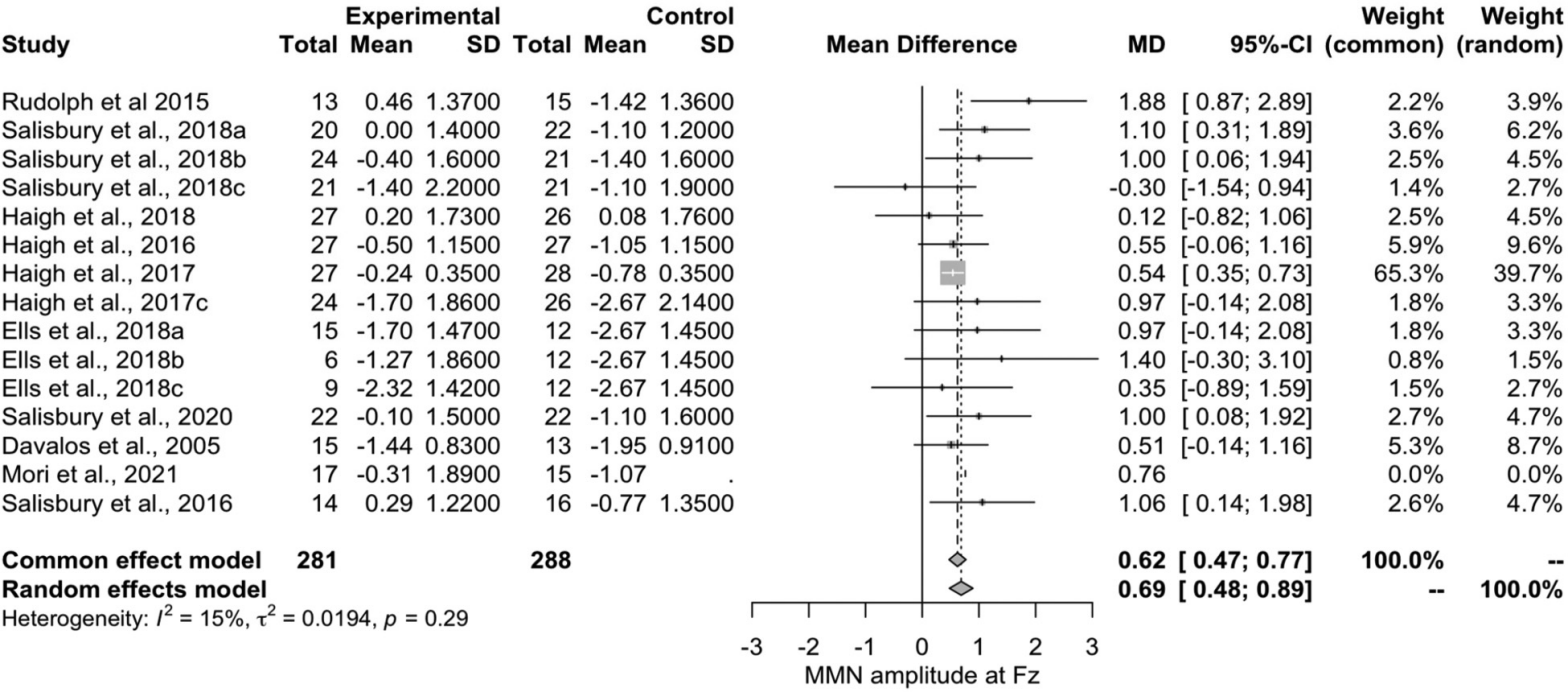
Forest plot of the 15 papers included in the meta-analysis.

One study^46^ had to be excluded from the meta-analysis and generation of the forest plots due to missing means and standard deviations. Results from the meta-analysis of the remaining 15 observations indicated that individuals with SZ exhibit amplitude deficits in response to the complex mismatch paradigms compared to controls, with small/moderate effect sizes (d = .47; Figure 2). Only two studies reported larger amplitudes in individuals with SZ compared to controls,^39,45^ both utilizing an ascending pitch paradigm. However, one of these studies^45^ specifically included clinical participants within the first 5 years of their illness, known as early phase psychosis. Three other studies^31,48,49^ also included individuals within the early phase of psychosis and the findings were consistent with the overall finding of decreased amplitudes for clinical participants compared to controls.

**Figure 3.**
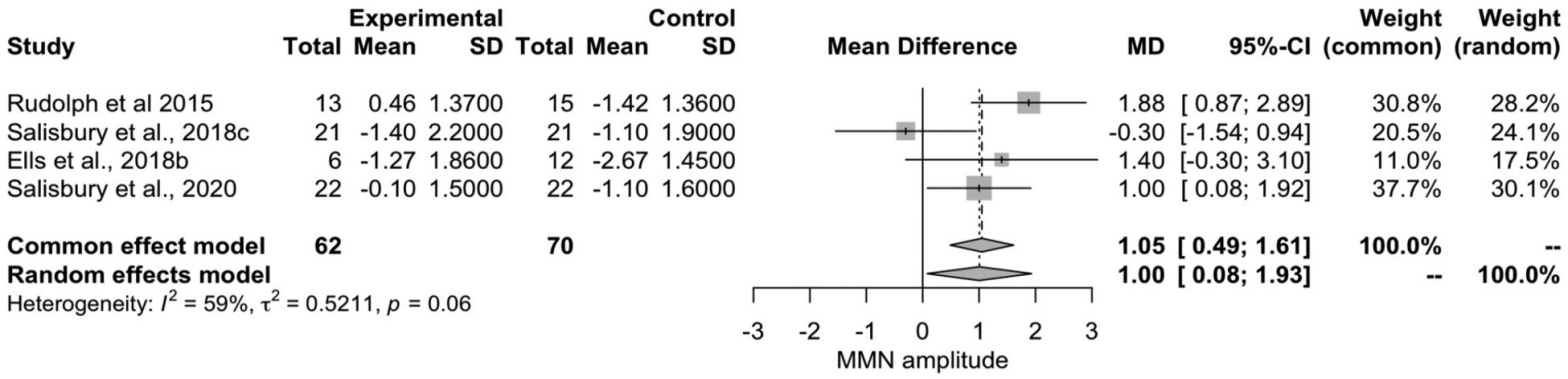
Forest plot of the four studies including early phase psychosis.

### Early Phase Psychosis Meta-Analysis

To explore the relationship between early phase psychosis and cMMN, we conducted a meta-analysis (*N *= 4^31,45,48,49^) with only those papers including individuals within the first 5 years of their diagnosis (*n *= 136, *n_EPP _*= 62^50,51^; Figure 3). Demographics were similar to what was reflected in the overall sample with fewer female participants (n = 44, clinical sample (n = 20) compared to males (n = 86; clinical sample (n = 42), missing data n = 2).

From this analysis, we observed that individuals in the early phase of psychosis have decreased amplitudes in response to cMMN paradigms compared to controls, with moderate/large effect sizes (d = .58) compared to the small/moderate effect size shown in established SZ.

### Quality Assessment

**Figure 4.**
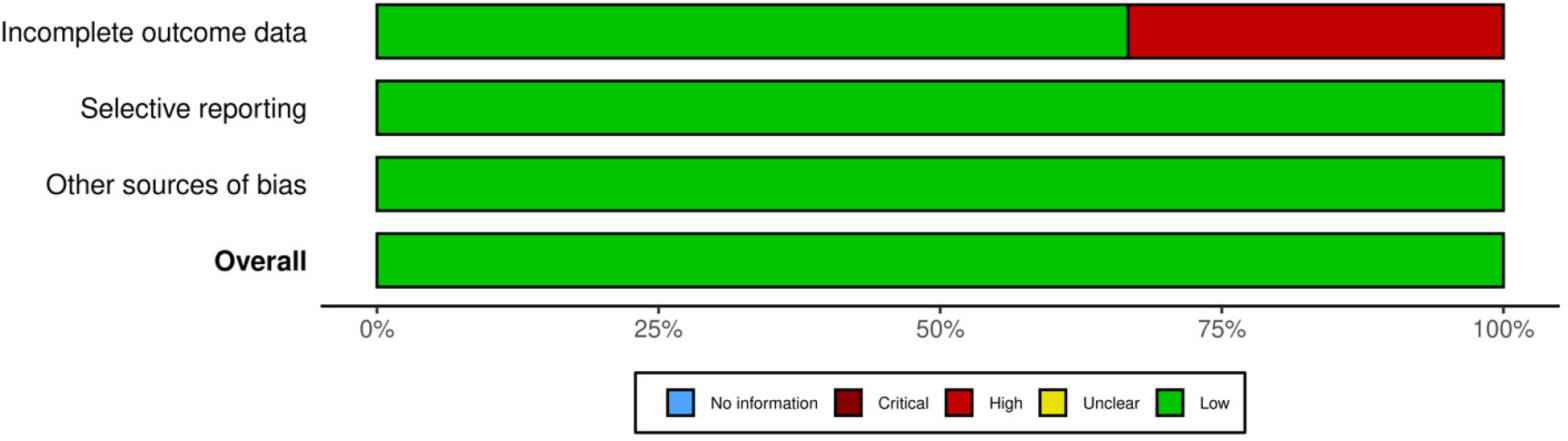
Quality assessment depicted visually, this image represents the quality of articles included in the meta-analysis. This was conducted using the Cochrane Risk of Bias scale. Figure was generated with Robvis.

The manuscripts examined showed an overall low risk of bias (refer to Figure 4).

## Discussion

We identified 11 unique studies utilizing five different cMMN paradigms. Within these 11 samples, we discovered five unique sub-studies that included separate participants or different paradigms; these sub-studies were then added to our analysis, resulting in a total of 16 studies. One study had to be excluded at the analysis phase as we were unable to extract means and standard deviations. Our analysis found that individuals with a primary diagnosis of SZ exhibit decreased cMMN amplitudes, with a small/moderate effect size compared to controls. We also found that individuals in the early phase of psychosis have significant reductions in cMMN amplitudes compared to control participants with a moderate/large effect size. The results pertaining to chronic SZ suggest the added benefit of these paradigms in chronic SZ samples is minimal; however, the discoveries concerning the early phase of psychosis were particularly intriguing.

While the cMMN is indeed a valuable tool for assessing auditory processing deficits in chronic SZ, it is not primarily designed for this purpose. Previous research has demonstrated the traditional MMN paradigms are effective in capturing these deficits during the chronic stages of the illness, rendering a more complex paradigm unnecessary. The purported utility of the cMMN lies in its potential ability to detect early stages of SZ. Specifically, the cMMN may enable the identification of early phases of illness progression in individuals experiencing FEP, due to its putatively higher computational demands. Salisbury et al (2020) demonstrated this in a sample of FEP, where traditional MMN paradigms failed to show group differences, while the later cMMN response to a pattern deviant exhibited significant between groups differences, with FEP individuals displaying decreased cMMN amplitudes compared to controls. Our exploratory analysis, encompassing articles investigating both early-phase psychosis and first episode, indicated an overall moderate/large effect, larger than what was shown for the chronic SZ sample, suggesting the deficit in cMMN amplitudes may be more pronounced in early-phase psychosis compared to the chronic stages of the illness. This reverses the direction of the findings observed with the traditional MMN paradigms where the effect sizes were largest in chronic SZ relative to the early-phase psychosis samples.^8,11^ While our exploratory analysis examining the cMMN in early phase psychosis included a small number of studies and therefore is not conclusive, this does reinforce the need for future work in this area and speaks to the promise that the cMMN paradigms may have in the early phase psychosis.

It has been suggested that the decrease in cMMN amplitude observed in individuals in the early phase of the illness is potentially attributed to the heightened computational demands of pattern processing, which may tax the higher-order processing networks in the brain.^7,48^ While the specific neural networks used to process the cMMN have yet to be identified, the complexity of these paradigms suggests that these neural networks may differ from those used to process the traditional MMN paradigms.^22^ Consequently, these complex paradigms may be better suited for detecting subtle pathophysiological changes during the early stages of disease progression compared to the traditional MMN paradigms. When comparing our findings to previous meta-analyses that examined traditional MMN paradigms in the first episode, we observe a more moderate/large effect size for the cMMN paradigm (d = .58) compared to the traditional paradigms, which exhibited more small/moderate effect sizes (d = .42^8^; d = .47^11^). Additionally, within our meta-analysis, we saw that the magnitude of the effect of the cMMN was larger on those in the early phase of psychosis (d = .58), compared to those in chronic SZ (d = .47). These results lend support to the notion that the cMMN may be a more sensitive method for detecting cognitive deficits in individuals during the early phases of illness progression, compared to the traditional paradigms. One difference between our sample and that of Erickson et al (2016) and Haigh et al (2017) is that they focused on individuals experiencing their first episode of psychosis, whereas we included all individuals in the early phase of psychosis (within the first 5 years of diagnosis^50,51^), with some studies also encompassing the first episode. Future research should strive to further understand the relationship between the cMMN and early-phase psychosis/FEP, to determine the utility of this paradigm in detecting the early stages of illness progression.

Our findings in chronic SZ align with previous studies on traditional MMN paradigms,^7,8,10^ which demonstrated decreased MMN amplitudes in those with SZ with a moderate effect size. Our findings were also similar Avissar et al’s (2018) findings from their analysis of complex abstract and pattern MMN stimuli. While Avissar et al (2018) reported moderate to large effect sizes (d = .75) on what they identified as complex abstract/pattern paradigms, not all of these paradigms meet our criteria for a complex paradigm and therefore a direct comparison is difficult. One key difference between our meta-analysis and that conducted by Avissar et al (2018) is that we specifically included complex auditory paradigms that involved the creation and subsequent disruption of a predetermined pattern such as that employed by Rudolph et al (2015) whereby a tone was missing from a pre-established pattern of tones. In contrast, Avissar et al (2018) included any paradigm with complex sensory stimuli that impacted multiple sensory dimensions, like that shown by Hamilton et al 2022 where they used a traditional oddball paradigm with a double deviant (ie, complex stimuli). Avissar et al (2018) also included paradigms using a visual oddball task which was not included in our analysis. Out of the five papers examined by Avissar et al (2018) as complex abstract/pattern deviants, only two meet the criteria that were established for this review. Nonetheless, the effect size reported by Avissar et al (2018) aligns with the observed in our meta-analysis overall (d = 0.58, vs d = 0.75). Suggesting that the complex MMN, whether it be elicited by a complex stimulus or violation of a pattern, is reduced in the SZ spectrum. Our findings tentatively suggest the cMMN elicited by a pattern violation yields a particularly large effect in early phase psychosis. It remains to be seen if similar patterns can be seen with the complex MMN elicited by complex stimuli; however, studies involving phonetic stimuli (that are inherently complex) suggest a small effect.^52^

We identified two studies^39,45^ that presented findings contradicting the overall effect observed in our analysis. These studies suggested that ascending pitch paradigms may not be as effective as the other cMMN paradigms. Salisbury et al (2018) propose that the reduced cMMN size in their study may be attributed to the complexity of their cMMN paradigm producing reductions in amplitude in both the clinical and control samples. While both this study^45^ and the study by Haigh et al (2018) report significant differences in cMMN amplitude between individuals with SZ and controls, their findings do not exhibit the same magnitude as the other studies included in this review; whether this is due to the activation of different neural networks, reduced activation of MMN generators, or some other mechanism is not known. Ultimately, most participants involved in this meta-analysis were using some type of medication, particularly atypical anti-psychotics, throughout the study period. Although there hasn’t been specific research on the effect of atypical antipsychotic medication on cMMN amplitudes, studies have investigated the medication's influence on the MMN triggered by the traditional oddball paradigm.^53–55^ These studies found that medication does not seem to affect the MMN. However, it is important to note that we cannot fully exclude the possibility that medication status could alter these outcomes. Additionally, participants had varying durations of illness, making it challenging to definitively determine the impact of illness duration on cMMN amplitude, future work should aim to better understand this relationship.

### Future Directions

We emphasize the infancy of this approach throughout the meta-analysis highlighting the need for more work to be done including individuals within the first 5 years of psychosis, which is considered to be a critical period of time for intervention.^50,51^ Additionally, more work needs to be done focusing on how the cMMN may differ with individuals experiencing psychotic symptoms who do not have a diagnosis of SZ. Specifically, work should be done to see if the results are the same in individuals with BD, or within a healthy control population that has experienced symptoms of psychosis, such as auditory hallucinations, as these findings may be symptom specific and be related to the positive symptoms associated with SZ as opposed to the disorder as a whole. It could be that these deficits in cMMN amplitude are symptom specific as opposed to illness-specific; however, with the current state of the literature, we are unable to investigate this further. Additionally, it is unclear if similar results are shown across males and females, or if the cMMN is related to age-related changes like we see with the traditional MMN^56,57^; the findings for these variables were not reported for all studies and thus, an analysis considering biological sex and age could not be performed. Future work should aim to include equal numbers of males and females (and include sex-stratified comparisons) in their statistical analyses and include a broad age range of participants to better understand how the cMMN may differ in individuals with psychosis when considering biological sex, and age.

To better understand if the cMMN paradigms evaluated in our meta-analysis are superior to the traditional oddball paradigms and the oddball paradigms incorporating complex stimuli, a subsequent meta-analysis would be beneficial in exploring this relationship further.

This meta-analysis further highlights the challenges involved in using and interpreting cMMN paradigms. In our review, we encountered five distinct paradigms; however, there are numerous ways to construct the cMMN paradigms that will elicit similar brain responses. Consequently, it becomes difficult to determine whether all these patterns are indexing the same neural pathways or representing slight variations, which could account for data variability. One potential approach to better understand these paradigms is through source localization analysis. Through this analysis, we have successfully identified the generators of the MMN using the traditional paradigm, which are located in the primary auditory cotex,^25,58,59^ as well as the frontal regions of the brain.^23,26^ However, similar analyses have not been conducted for the cMMN paradigms, therefore we are unsure where the specific neural generators are and whether they vary depending on the paradigm used. Future studies should aim to explore this relationship by conducting source localization analysis and investigating the neural generators associated with the various cMMN paradigms.

Finally, magnetic resonance spectroscopy (MRS) is another methodology that can contribute to understanding the cMMN paradigms and their neural generators. Previous studies have demonstrated the crucial involvement and dependence of MMN generation, based on traditional paradigms, on N-methyl-D-aspartate (NMDA) receptors.^60,61^ This linkage connects the MMN response to the glutamatergic system in the brain, which is also impacted by psychosis.^62^ Previous research has demonstrated this link by administering competitive and non-competitive NMDA receptor antagonists, which inhibit the generation of the MMN from traditional paradigms.^63–65^ While we assume the cMMN is also dependent on these NMDA receptors, to date, no study has examined this relationship. Future research should aim to identify the neural generators of the cMMN and determine the most effective paradigm(s) to elicit these responses. By doing so, we will be better equipped with the necessary information to accurately evaluate the impact of SZ and early phase psychosis on cMMN amplitudes. Incorporating MRS into such studies can provide valuable insights into the neurochemical mechanisms underlying the cMMN paradigm that EEG alone does not provide.

## Conclusions

Overall, we found that individuals with SZ show amplitude decreases on cMMN-eliciting paradigms. An exploratory analysis suggests that this deficit may be more pronounced in individuals within the first 5 years of illness progression. This supports the theory that the cMMN may be a useful biomarker for detecting the early phases of SZ and psychosis.
